# 

**Published:** 1903-03-21

**Authors:** 


					the hospital. ? The standard of highest purity."?Lancet. maech 21st, 1903.
CADBURY'S CADBURY'S
cocoa y i\ Jinil m cocoa
MO DRUQS OR ALKALIES
* \s\)\*-rkny
No. 860. Vol. XXXIII. iJSSSJi) Saturday,
March 21st, 1903.
[SubSCgriptlonTerms,-J pRI0H TWOPENCE.

				

